# Glioma Association and Balancing Selection of *ZFPM2*

**DOI:** 10.1371/journal.pone.0133003

**Published:** 2015-07-24

**Authors:** Shui-Ying Tsang, Lingling Mei, Weiqing Wan, Jun Li, Yi Li, Cunyou Zhao, Xiaofan Ding, Frank W. Pun, Xiaoxia Hu, Jianmin Wang, Junyi Zhang, Rongcheng Luo, Siu-Tim Cheung, Gilberto K. K. Leung, Wai-Sang Poon, Ho-Keung Ng, Liwei Zhang, Hong Xue

**Affiliations:** 1 Division of Life Science and Applied Genomics Centre, Hong Kong University of Science and Technology, Clear Water Bay, Hong Kong, China; 2 Department of Neurosurgery, Beijing Tiantan Hospital, Capital Medical University, Beijing, China; 3 Division of Neurosurgery, Department of Surgery, Prince of Wales Hospital, Chinese University of Hong Kong, Shatin, Hong Kong, China; 4 Department of Hematology, Institute of Hematology, PLA, Changhai Hospital, Second Military Medical University, Shanghai, China; 5 Cancer Center, Nanfang Hospital, Southern Medical University, Guangzhou, China; 6 Division of Neurosurgery, Department of Surgery, Li Ka Shing Faculty of Medicine, The University of Hong Kong, Queen Mary Hospital, Hong Kong, China; 7 Department of Anatomical and Cellular Pathology, Prince of Wales Hospital, Chinese University of Hong Kong, Shatin, Hong Kong, China; University of Alabama at Birmingham, UNITED STATES

## Abstract

*ZFPM2*, encoding a zinc finger protein and abundantly expressed in the brain, uterus and smooth muscles, plays important roles in cardiac and gonadal development. Abnormal expression of *ZFPM2* in ovarian tumors and neuroblastoma has been reported but hitherto its genetic association with cancer and effects on gliomas have not been studied. In the present study, the hexamer insertion-deletion polymorphism rs71305152, located within a large haplotype block spanning intron 1 to intron 3 of *ZFPM2*, was genotyped in Chinese cohorts of glioma (n = 350), non-glioma cancer (n = 354) and healthy control (n = 463) by direct sequencing and length polymorphism in gel electrophoresis, and *ZFPM2* expression in glioma tissues (n = 69) of different grades was quantified by real-time RT-PCR. Moreover, potential natural selection pressure acting on the gene was investigated. Disease-association analysis showed that the overall genotype of rs71305152 was significantly associated with gliomas (*P* = 0.016), and the heterozygous genotype compared to the combined homozygous genotypes was less frequent in gliomas than in controls (*P* = 0.005) or non-glioma cancers (*P* = 0.020). *ZFPM2* mRNA expression was negatively correlated with the grades of gliomas (*P* = 0.002), with higher expression levels in the low-grade gliomas. In the astrocytoma subtype, higher *ZFPM2* expression was also correlated with the rs71305152 heterozygous genotype (*P* = 0.028). In addition, summary statistics tests gave highly positive values, demonstrating that the gene is under the influence of balancing selection. These findings suggest that *ZFPM2* is a glioma susceptibility gene, its genotype and expression showing associations with incidence and severity, respectively. Moreover, the balancing selection acting on *ZFPM2* may be related to the important roles it has to play in multiple organ development or associated disease etiology.

## Introduction

The zinc finger protein multitype 2 (*ZFPM2*) gene, also known as friend of GATA-2 (*FOG2*), encodes a transcriptional cofactor of members of the GATA-binding family that regulates expression of key genes essential for the development of multiple organs [1]. By interacting with GATA factors, ZFPM2 modulates this regulatory activity, and is known to play important roles in cardiac, gonadal, and pulmonary development [1–4].

Previously, *ZFPM2* has been found to be involved in the pathogenesis of cancers, e.g. its abnormal gene expression in sex cord-derived ovarian tumors [5] and neuroblastoma [6]. Moreover, the effect of *ZFPM2* on cell differentiation [7] and apoptosis [8] are suggestive of a tumor suppressor role in cancers. However, there have been no genetic association studies and the significance of *ZFPM2* in gliomas is unclear. Gliomas, which attack the brain and spine, are the most common and malignant primary tumor in the central nervous system [9–11]. The molecular characteristics of glioma subtypes have been extensively investigated in relation to genetic heterogeneity or aberrant gene expression [12–15]. However, the number of explicit glioma susceptibility genes among the ~30,000 human genes [16] is limited based on previous genome-wide or selected gene association studies. So far *TERT* [17–20], *RTEL1* [17,19,21], *PHLDB1* [17,19], *EGFR* [22,23], *ORMDL3* [24], *CLPTM1L* [20], *H2AFX* [25], *VEGFA* [26] and *GSTP1* [18] have been reported as glioma associated genes in *Han* Chinese and other populations. The importance of zinc finger proteins in cancer etiology is well established, and since *ZFPM2* is abundantly expressed in premature and adult brain, cooperating with GATA factors to regulate neural gene expression and development [1], analysis of *ZFPM2* in gliomas of different grades may shed light on its potential relationship with glioma risk.

In view of the important biological roles played by *ZFPM2*, natural selection likely contributes to its patterns of genetic variations. Balancing selection is proposed as a major mechanism for maintaining phenotypic and genetic variation in natural populations [27], and it has been invoked to explain genetic variation in several human genes, including genes in the immune response [28] as well as in cancer [29].

In the present study, the hexamer insertion-deletion polymorphism rs71305152 was genotyped in glioma and control cohorts, as well as cohorts of non-glioma cancer in order to estimate the contribution of rs71305152 to cancer risk, especially for gliomas, and the evolutionary pressure acting on *ZFPM2* was investigated. The indel resides within a large haplotype block so can act as a tagging marker and, relative to single nucleotide markers, it can be more accurately detected. Disease-association results showed that rs71305152 was associated with gliomas at the genotype level, suggesting that *ZFPM2* represents a glioma susceptibility gene. Moreover, *ZFPM2* could be a useful disease severity indicator, as its expression levels were negatively correlated with glioma grades, and summary statistics tests demonstrated that the gene is under the influence of balancing selection.

## Methods

### Ethics Statement

Written informed consent was obtained from each participant. Subject recruitment and sample collection were approved by the research ethics review boards of Prince of Wales Hospital and Queen Mary Hospital in Hong Kong, and Beijing Tiantan Hospital, Shanghai Changhai Hospital and Guangzhou Nanfang Hospital in China.

### Study cohorts

The various cohorts in this study were enrolled from Beijing, Shanghai, Guangzhou and Hong Kong. The glioma cohort were unrelated Chinese *Han* patients recruited from Prince of Wales Hospital and Queen Mary Hospital in Hong Kong, and Beijing Tiantan Hospital. Patients were diagnosed based on surgical pathological records, and classified into four subgroups according to WHO classification [11,30], namely low-grade astrocytomas (A II), high-grade astrocytomas (A III—IV); low-grade oligodendroglial tumors (grade II oligodendrogliomas and oligoastrocytomas, O + OA II); high-grade oligodendroglial tumors (anaplastic oligodendrogliomas and anaplastic oligoastrocytomas, AO + AOA III). *ZFPM2* expression was analyzed in 69 of the glioma patients (age, 43.6 ± 15.9 year old; 40 males and 29 females). The control cohort consisted of healthy volunteers recruited by Hong Kong Red Cross, and Beijing volunteers. Leukemia, lymphoma and lung cancer cohorts were unrelated Chinese *Han* individuals recruited from Shanghai Changhai Hospital and Guangzhou Nanfang Hospital. The demographic characterizations of all the samples are described in S1 Table.

### DNA and RNA Samples

Peripheral white blood cells, formalin-fixed paraffin-embedded (FFPE) glioma tissues, and fresh glioma tissues were collected for DNA and/or RNA extraction. Glioma U87 cells (provided by Prince of Wales Hospital) were harvested for RNA extraction. DNA was extracted from 5 ml peripheral blood by the phenol-chloroform method. DNA was extracted from FFPE samples with xylene, PCR buffer and Proteinase K, and mRNA was isolated from ~100 mg samples of frozen glioma tissue or glioma U87 cells with TRIzol solution (Invitrogen).

### PCR of fragment containing rs71305152

An insertion-deletion polymorphism locus (rs71305152, TTTTCT/–), previously amplified by the AluScan method [31] and verified by Sanger sequencing from the paired DNA of tumor tissue and peripheral white blood cell of an anaplastic oligodendroglioma patient, was employed as genotyping marker.

Nested PCR was used to amplify the gene fragment containing rs71305152. For this purpose two pairs of PCR primers were designed, and their specificities were checked with PerlPrimer software version 1.1.19. The fragment size of the first PCR was 1,114 bp, and was amplified using the forward primer ‘5-TAAAGCAGCTGTCAGATCACATCC-3’ and reverse primer ‘5-AAGTTATTGTGCAGGAACATGGC-3’. Each 15 μl PCR reaction mixture contained 1.5 μl 10x long PCR buffer (500 mM Tris-Cl, pH 9.0, 160 mM ammonium sulfate, 25 mM MgCl_2_, 1.5 mg/ml bovine serum albumin), 4 mM MgCl_2_, 20 μM dNTP mix, 0.1 μM forward primer and reverse primer, 0.5 unit *Taq* polymerase and 40–100 ng genome DNA. The PCR amplification was performed at 95°C 5 min, 35 cycles of 95°C 30 s, 69.3°C 30 s, and 72°C 1.5 min, and finally another 5 min at 72°C.

The second PCR reaction was performed with 0.1 μM forward primer ‘5-GTCGACTTTGACGGTAATGTCCT-3’ and reverse primer ‘5-GAGGTAAGAGTATAATCCAGAAGAC-3’ contained in 15 μl PCR mixture, which also included 1.5μl 10 x long PCR buffer, 4 mM MgCl_2_, 20 μM dNTP mix, 0.5 unit *Taq* polymerase and 3 μl purified first PCR products. PCR amplification was conducted at 95°C 5 min, 35 cycles of 95°C 30 s, 67°C 30 s, and 72°C 1.5 min, and finally another 5 min at 72°C. For each sample, 3 μl PCR products were mixed with 2 μl 6x DNA loading dye and loaded on 0.7% ethidium bromide stained agarose gel in 1x Tris-acetate-EDTA (TAE) buffer. Following electrophoresis, the presence of products of 1,009 bp was confirmed under UV.

### Genotyping by gel electrophoresis of PCR products

In order to distinguish between the three rs71305152 genotypes by gel electrophoresis, PCR was performed with the forward primer ‘5-AAAATTTTCATCTTGA-3’ and reverse primer ‘5- ATTCTCATCCCGTAT -3’ to amplify a 44-bp fragment in *ZFPM2*. In each instance, 15 μl PCR reaction was conducted containing 0.1 μM of each primer, 1.5 μl 10x long PCR buffer, 4 mM MgCl_2_, 20 μM dNTP mix, 0.5 unit *Taq* polymerase and 100 ng DNA. PCR amplification was carried out at 95°C 5 min, 35 cycles of 95°C 30 s, 51°C 20 s, and 72°C 10 s, and finally another 3 min at 72°C. PCR products (10 μl) of each sample were mixed with 2 μl 6x DNA loading dye and loaded on 5% ethidium bromide stained agarose gel in 1x TAE buffer. Following electrophoresis, the presence of the 44-bp product was confirmed under UV.

### Genotyping by direct sequencing of PCR products

The genotypes of rs71305152 for each sample were also identified by direct DNA sequencing. Each 15 μl sequencing reaction contained 0.75 μl BigDye Terminator v3.1 (Applied Biosystems), 3 μl BigDye Terminator v3.1 5xSequencing Buffer (Applied Biosystems), 0.24 μM sequencing primer ‘5-GCTGTTAAATGTGTATACTT-3’, and 3 μl purified PCR products. Reaction was performed at 96°C 1 min, followed by 25 cycles of 96°C 10 s, 50°C 5 s, 60°C 4 min. The sequencing was carried out using the 3130*xl* Genetic Analyzer, and the three different genotypes were determined using the BioEdit biological sequence alignment editor software version 7.0.5.

### Quantitation of *ZFPM2* expression

*ZFPM2* expression levels in glioma tissue were measured by real-time quantitative reverse transcription PCR (real-time qRT-PCR). Total RNA was firstly reverse transcribed to cDNA through reverse transcription-polymerase chain reaction (RT-PCR) with TaqMan Reverse Transcription Kit (Applied Biosystems). Each 20 μl RT-PCR contained 2 μl 10x RT buffer, 2 μl 10x RT Random Primers, 80 mM 25x dNTP Mix, 1 unit MultiScribe Reverse Transcriptase (Applied Biosystems), 40 unit RNaseOUT Recombinant Ribonuclease Inhibitor (Invitrogen) and 1 μg mRNA. RT-PCR was carried out at 25°C 10 min, 37°C 120 min, and finally 85°C 5 min. Real-time qRT-PCR was subsequently performed with two housekeeping genes hypoxanthine phosphoribosyltransferase 1 (*HTRP1*) and TATA box binding protein (*TBP*), which are appropriate for glioma tissue gene expression analysis [32]. The *HTRP1* and *TBP* primers were as described [32]. The *ZFPM2* forward primer ‘5-ATTCTTTGAAGACAAAGGCTCAG-3’ within exon 5 and reverse primer ‘5-GGTACATCCCTTCTGTGAGAG-3’ within exon 7 were designed using the PerlPrimer software version 1.1.19. Each 10 μl Real-Time qRT-PCR mixture contained 0.1 μM per primer, 4 μl SYBR *Premix Ex Taq* (2x) (TAKARA), 0.2 μl ROX Reference Dye II (50x), and 0.04 μg sample RNA cDNA product. The reaction was performed on the 7500 Fast Real-Time PCR System (Applied Biosystem) at 95°C 30 s, 40 cycles of 95°C 3 s, 60°C 30 s, and one cycle of 95°C 15 s, 60°C 1 min, 95°C 15 s. The *ZFPM2* expression levels were determined from C_T_ values, and batch-to-batch experimental variations correction was carried out utilizing mRNA of U87 cells as calibrator. The expression levels were further normalized against the geometric mean of the expression levels of the two housekeeping genes described above.

### Statistical analysis

The statistical tests were regarded as significant when *P* value was less than 0.05. The genotype frequency deviation from Hardy-Weinberg equilibrium was tested based on Pearson’s Chi-square test through the online program (http://ihg.gsf.de/cgi-bin/hw/hwa1.pl). Pearson’s Chi-square test in the program UNPHASED version 3.1.2 was employed for analyzing overall allele and genotype frequency difference between the case and control cohorts. The same test in SPSS version 17.0 (SPSS Inc., Chicago, IL, USA) was employed for comparison of the frequency difference between the case and control cohorts for each genotype sub-group. Student’s t-test and ANOVA in SPSS version 17.0 were used to test the significance of correlation of the three glioma grades, survival status, or genotypes with *ZFPM2* mRNA expression levels in the glioma tissues. Kaplan-Meier non-parametric test [33] and Cox regression in SPSS version 17.0 was used to generate survival curves to evaluate the importance of rs71305152 genotypes to survival time.

### Summary statistics

Phased haplotype data for the *ZFPM2* gene (genomic region 106400 kb—106886 kb) on chromosome 8 was retrieved from Phase III of the HapMap Project and used as input for the program DnaSP [34] to yield the summary statistics Tajima’s D [35], Fu and Li’s D* and Fu and Li’s F* [36] for each of the 11 populations surveyed in this phase of the HapMap Project. These statistics were further analyzed to determine whether they were significantly different from expected values under a standard neutral model by performing coalescent simulation assuming no recombination.

## Results

### Genotypic correlation of rs71305152 with gliomas

**Fig 1:**
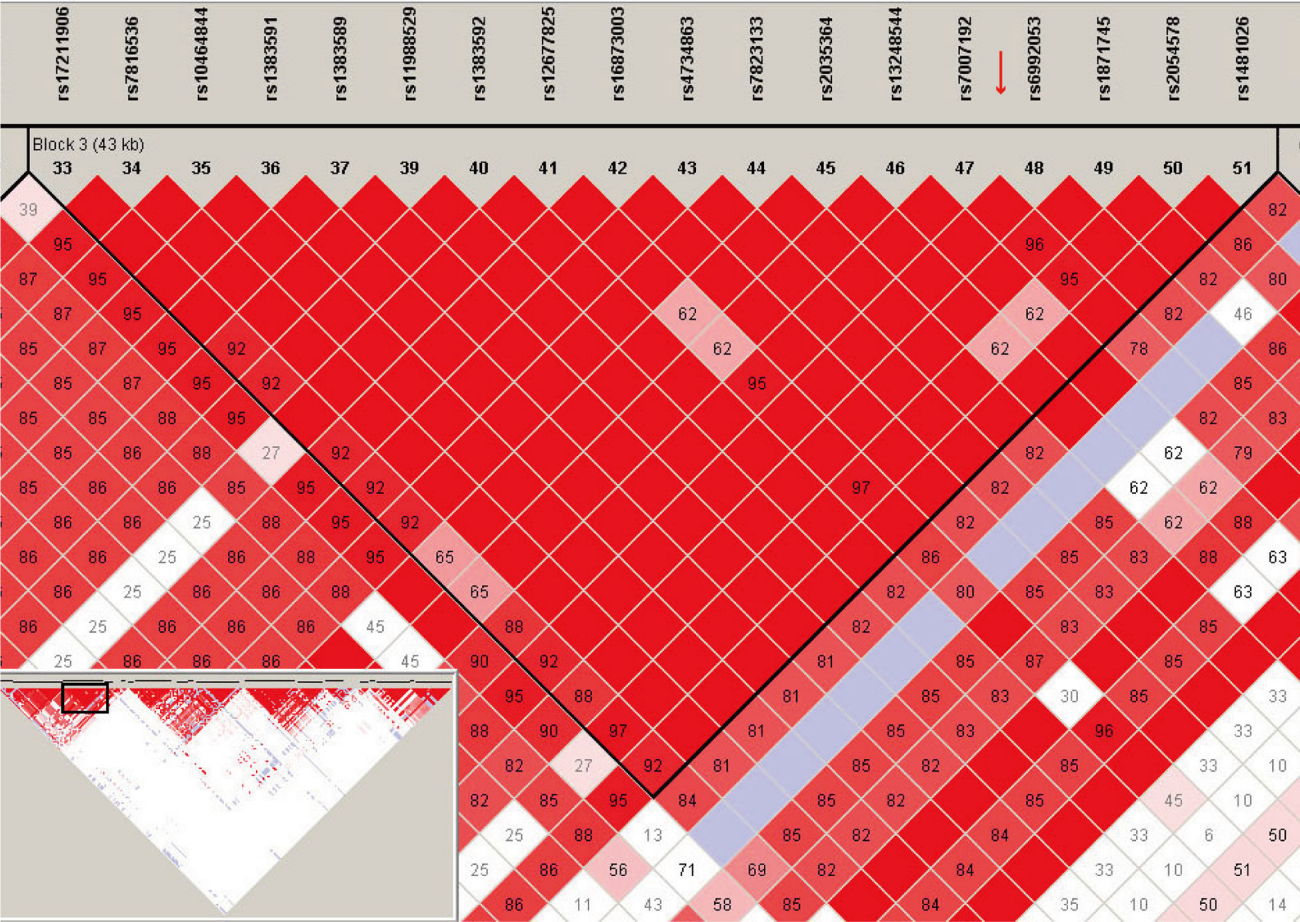
Linkage disequilibrium (LD) plot generated using Haploview. The plot depicts the haplotype block containing the rs71305152 indel for the HapMap CHB (Han Chinese in Beijing) population. The rs numbers of featured SNPs are given above the plot and the red arrow indicates the location of the rs71305152 indel. The LD plot for the whole *ZFPM2* gene is given in the inset and the box within the inset indicates the depicted, enlarged region. The standard Haploview LD color scheme based on D’ and LOD (log of the likelihood odds ratio) is used and the value of 100 x D’ for each SNP pair is given in its respective tile unless D’ = 1.

**Fig 2:**
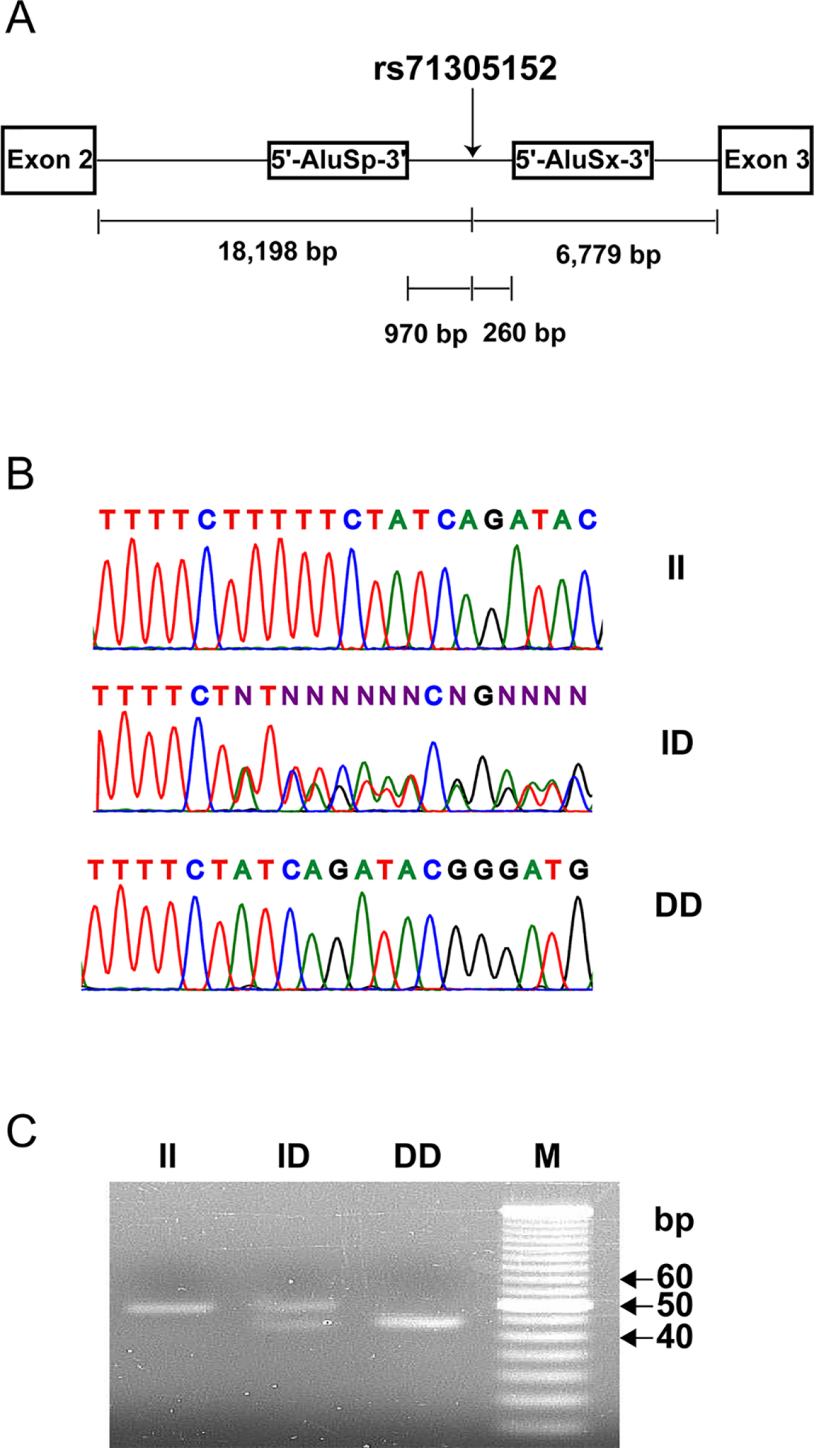
The hexamer insertion-deletion polymorphism rs71305152 in *ZFPM2*. (A) Schematic diagram showing that rs71305152 is located within intron 2 of *ZFPM2*, between two neighboring head to tail oriented AluSx and AluSp elements. The distance of rs71305152 to each exon and Alu element is indicated. (B) and (C) show the three genotypes of homozygous insertion (II), heterozygous (ID) and homozygous deletion (DD) identified by direct DNA sequencing and gel electrophoresis, respectively. M denotes 5-bp DNA size marker.

The polymorphism marker rs71305152 resides within a highly-linked haplotype block spanning over 40 kb in *ZFPM2* (Fig 1), and is located at 18,198 bp downstream of exon 2 and 6,779 bp upstream of exon 3, between a pair of Alu elements (Fig 2A). The three genotypes of rs71305152, namely the double insertion or II (TTTTCT/TTTTCT), heterozygous or ID (TTTTCT/–), and double deletion or DD (–/–) genotypes, can be readily detected through both direct sequencing (Fig 2B) and gel electrophoresis (Fig 2C).

All genotype frequencies within study cohorts did not deviate from Hardy-Weinberg equilibrium with exception of the glioma cohort (*P* = 0.0015), according to the Hardy-Weinberg equilibrium test. In addition, there was no statistical difference in genotype frequencies between the control cohorts from two different geographical regions, Hong Kong and Beijing. The allele frequency difference was analyzed between different cancer-control cohorts, and neither insertion allele nor deletion allele was found to be associated with any type of cancer (Tables 1 and 2). At the genotype level, the heterozygous genotype frequency was extensively decreased in the glioma cohort compared to the control cohort (*P* = 0.016) (Table 1). The inclusion of gender as covariate did not affect this association. However, although the genotype frequency difference between the glioma cohort and the combined non-glioma cancer cohort was not statistically significant (Table 1), no genotype association was found in the non-glioma cancer types (Table 2).

**Table 1:** Allele and overall genotype association of rs71305152 with glioma.

Cohort	Subject	Allele					Genotype			
	n (M/F Ratio*^b^*)	I % (n)	D % (n)	OR*^c^*	95% CI*^d^*	*P* *^e^*	II % (n)	ID % (n)	DD % (n)	*P* *^f^*
Glioma	350 (1.54)	33.57 (235)	66.43 (465)				15.14 (53)	36.86 (129)	48.00 (168)	
Non-glioma*^a^*	354 (1.51)	35.45 (251)	64.55 (457)	0.920	0.739–1.146	0.458	12.71 (45)	45.48 (161)	41.81 (148)	0.066
Control	463(1.02)	34.77 (322)	65.23 (604)	0.948	0.770–1.166	0.613	11.45 (53)	46.65 (216)	41.90 (194)	**0.016**

^*a*^ Non-glioma cohort includes lung cancer, leukemia and lymphoma cohorts;

^*b*^ M/F Ratio is the ratio of successfully genotyped male to female;

^*c*^ OR is the odds ratio;

^*d*^ 95% CI is 95% confidence interval;

^*e*^ *P* values pertain to allele frequency difference between glioma cohort and either non-glioma cancer cohort or control cohort;

^*f*^ *P* values pertain to overall genotype frequency difference between glioma cohort and either non-glioma cancer cohort or control cohort;

*P* value of less than 0.05 is highlighted.

**Table 2:** Allele and overall genotype association of rs71305152 with individual non-glioma cancers.

Cohort	Subject	Allele					Genotype			
	n (M/F Ratio*^a^*)	I % (n)	D % (n)	OR*^b^*	95% CI*^c^*	*P* *^d^*	II % (n)	ID % (n)	DD % (n)	*P* *^e^*
Lung Cancer	109 (2.11)	38.99 (85)	61.01 (133)	1.157	0.838–1.597	0.377	15.60 (17)	46.79 (51)	37.61 (41)	0.629
Leukemia	96 (1.18)	40.10 (77)	59.90 (115)	1.212	0.866–1.696	0.264	15.63 (15)	48.96 (47)	35.42 (34)	0.528
Lymphoma	149 (1.40)	29.87 (89)	70.13 (209)	0.771	0.570–1.043	0.089	8.73 (13)	42.28 (63)	48.99 (73)	0.228
Control*^f^*	281 (0.99)	35.59 (200)	64.41 (362)				12.10 (34)	46.98 (132)	40.93 (115)	

^*a*^ M/F Ratio is the ratio of successfully genotyped male to female;

^*b*^ OR is the odds ratio;

^*c*^ 95% CI is 95% confidence interval;

^*d*^ *P* values pertain to allele frequency difference between cancer cohort and control cohort;

^*e*^ *P* values pertain to overall genotype frequency difference between cancer cohort and control cohort;

^*f*^ Control cohort consisted of healthy individuals from Hong Kong.

**Fig 3:**
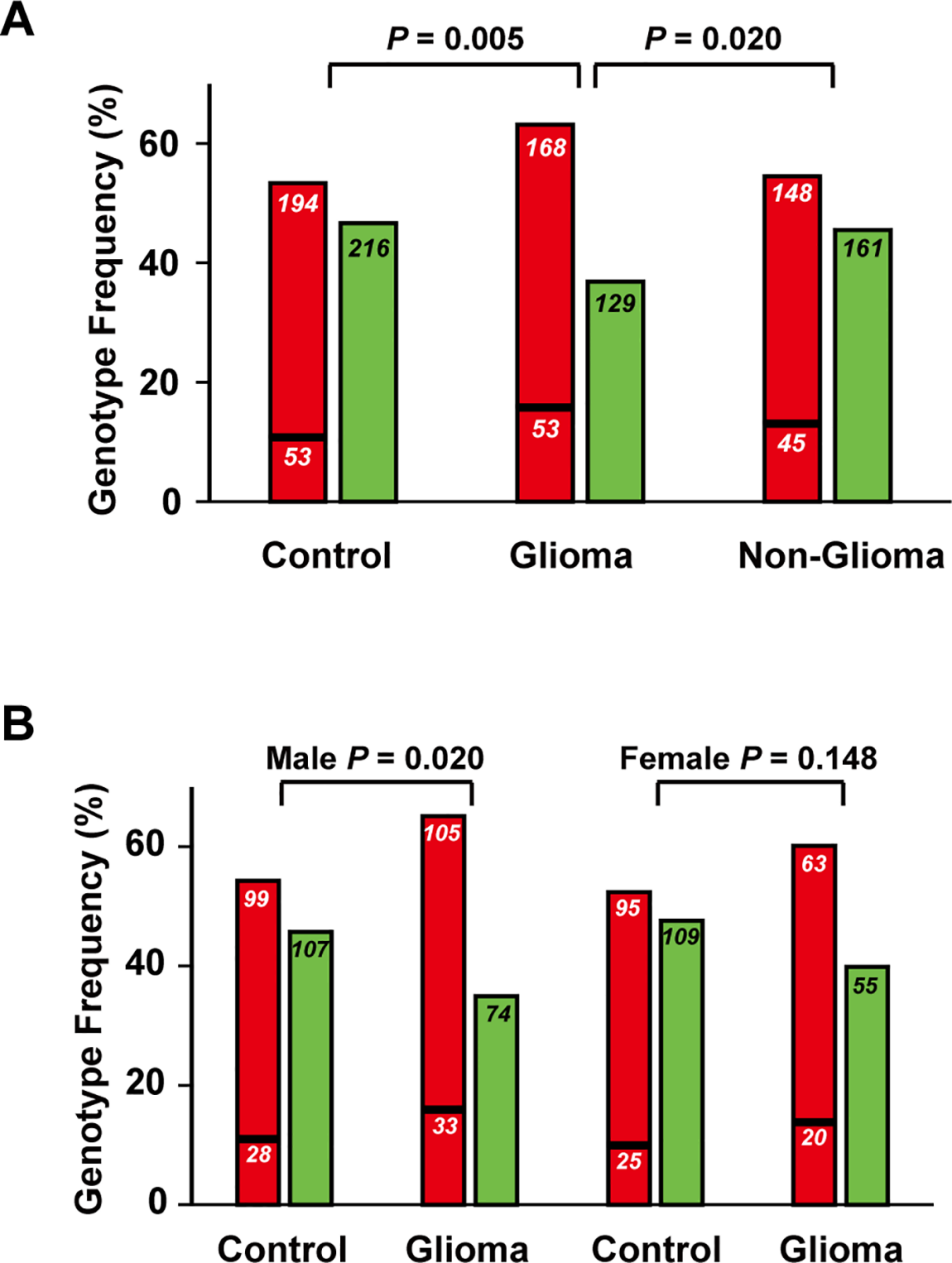
Association of rs71305152 genotypes with gliomas. In part (A), the heterozygous genotype was significantly decreased in the glioma cohort compared to either the control cohort (*P* = 0.005), or the non-glioma cancer cohort (*P* = 0.020). In part (B), the heterozygous genotype was only significantly decreased in the male glioma cohort (*P* = 0.020) but not in the female glioma cohort (*P* = 0.148) compared to control. Red bars represent the combined homozygous deletion (upper portion) and homozygous insertion (lower portion) genotypes, and green bars represent the heterozygous genotype. The sample size of each genotype group is shown within the bars.

Pearson’s Chi-square test was conducted as a post-hoc test on the frequency difference of any two genotypes between the glioma cohort on the one hand, and control cohort or non-glioma cancer cohort on the other. As shown in Table 3, in comparing the frequencies of the homozygous insertion (II) and heterozygous (ID) genotypes, the II frequency was more extensively increased in the glioma cohort compared to the control cohort (Test 2, OR = 1.675, 95% CI = 1.080–2.597, *P* = 0.021). In comparing the frequencies of the homozygous deletion (DD) and heterozygous ID genotypes, the DD frequency was significantly increased in the glioma cohort, relative to either the control cohort (Test 3, OR = 1.450, 95% CI = 1.074–1.958, *P* = 0.015), or the non-glioma cancer cohort (Test 6, OR = 1.416, 95% CI = 1.029–1.949, *P* = 0.033). Furthermore, in comparing the frequencies of the combined homozygous genotypes and the ID genotype, the ID frequency was substantially decreased in the glioma cohort relative to either the control cohort (*P* = 0.005) or the non-glioma cancer cohort (*P* = 0.020) (Fig 3A). When the genders were analyzed separately, as shown in Fig 3B, the frequency of the heterozygous genotype was significantly lower in male glioma patients relative to healthy controls (*P* = 0.020), but not in the female glioma patients (*P* = 0.148). As shown in S1 Fig, there was no significant genotype frequency difference between the four subtypes of gliomas, *viz*. A II (n = 44), A III—IV (n = 162), O + OA II (n = 89), and AO + AOA III (n = 55) (*P* = 0.956).

**Table 3:** Pearson’s Chi-square tests on pairwise genotype frequency difference between pairs of cohorts for rs71305152.

Test	Cohort 1			Cohort 2			OR*^a^*	95% CI*^b^*	*P* *^c^*
	Subject	Genotype	n	Subject	Genotype	n			
1	Glioma	II	53	Control	II	53	1.155	0.749–1.779	0.515
		DD	168		DD	194			
2	Glioma	II	53	Control	II	53	1.675	1.080–2.597	**0.021**
		ID	129		ID	216			
3	Glioma	DD	168	Control	DD	194	1.450	1.074–1.958	**0.015**
		ID	129		ID	216			
4	Glioma	II	53	Non-glioma Cancer	II	45	1.038	0.659–1.635	0.874
		DD	168		DD	148			
5	Glioma	II	53	Non-glioma Cancer	II	45	1.470	0.928–2.328	0.100
		ID	129		ID	161			
6	Glioma	DD	168	Non-glioma Cancer	DD	148	1.416	1.029–1.949	**0.033**
		ID	129		ID	161			

^*a*^ OR is odds ratio;

^*b*^ 95% CI is 95% confidence interval;

^*c*^ *P* values pertain to the frequency difference of any two genotypes between glioma cohort on the one hand, and control cohort or non-glioma cancer cohort on the other;

*P* values of less than 0.05 are highlighted.

### Correlation of *ZFPM2* expression with glioma grades

**Fig 4:**
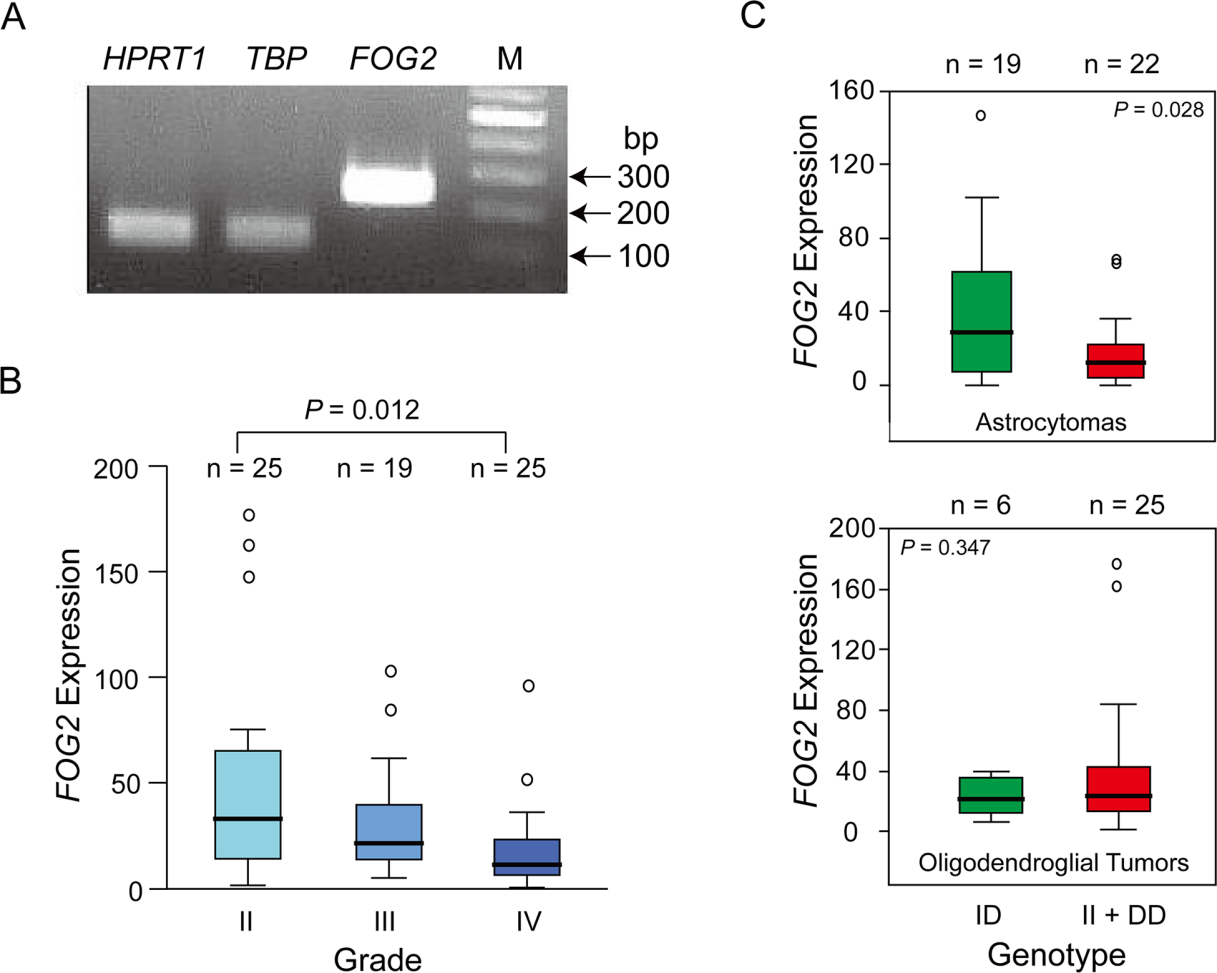
Correlation of *ZFPM2* expression with glioma grades and rs71305152 genotypes. (A) Gel electrophoresis of real-time qRT-PCR products of *ZFPM2* and the two housekeeping genes *HPRT1* and *TBP*. M denotes 100-bp DNA size marker. (B) Strong negative correlation between the *ZFPM2* expression and the three glioma grades (*P* = 0.006). (C) Correlation of *ZFPM2* expression with rs71305152 genotypes for astrocytomas (Upper panel; *P* = 0.028), and oligodendroglial tumors (Lower panel; *P* = 0.347), respectively. In parts B and C, the horizontal line in each box indicates the median; the upper and lower bounds of the box represent the 75th and 25th percentiles, respectively; the whiskers for each box mark either the values 1.5 times the interquartile range from the upper and lower edge of the box or the maximum and minimum values, whichever is the smaller; and the circles indicate outliers.

The *ZFPM2* mRNA expression levels of 69 glioma patients were examined using real-time qRT-PCR (Fig 4A), and the range and median levels of *ZFPM2* total mRNA for the different subtypes and grades of glioma are shown in Table 4. When ANOVA analysis was conducted in the combined-subtype cohort, significant difference was found between *ZFPM2* expression levels in the different glioma grades (*P* = 0.012) (Fig 4B). Post-hoc tests revealed that the *ZFPM2* expression in grade II tumors were significantly higher than that in grade IV tumors (*P* = 0.003). Moreover, there was no significant correlation found between *ZFPM2* expression with either age or gender (*P* = 0.406 for age; *P* = 0.244 for gender). When ANOVA was employed to compare the different grades in the individual glioma subtype, significant difference in *ZFPM2* expression between grades was found for astrocytoma (*P* = 0.006), but not for oligodendroglial tumors where grade IV tumors are absent (Table 4).

**Table 4:** Correlation of *ZFPM2* expression level with glioma grades.

Subtype	Grade*^a^*	n	Expression		
			Range	Median	*P* *^b^*
Astrocytomas	II	8	3.29–146.50	62.97	**0.006**
	III	5	4.81–102.60	42.17	
	IV	25	0.23–95.76	11.35	
Oligodendroglial Tumors*^c^*	II	17	1.41–176.30	24.18	0.263
	III	14	6.92–83.95	21.39	

^*a*^ Grade represents WHO classification of gliomas on the basis of histological features;

^*b*^ *P* values pertain to correlation between *ZFPM2* expression and glioma grades. *P* value of less than 0.05 is highlighted;

^*c*^ Oligodendroglial tumors include the subtypes, oligodendrogliomas and oligoastrocytomas.

To determine the possible association between genotypes and *ZFPM2* expression levels, the 69 patients were separated into two subgroups for analysis, those with homozygous (both II and DD) genotypes and those with the ID genotype. Significant difference in *ZFPM2* expression between the two genotype subgroups was observed for astrocytomas (n = 38, *P* = 0.028) as determined using Student’s t-test, with lower expression in the homozygous group, but no difference was found for oligodendroglial tumors (n = 31, *P* = 0.347) (Fig 4C).

### Correlation of *ZFPM2* expression with survival

**Fig 5:**
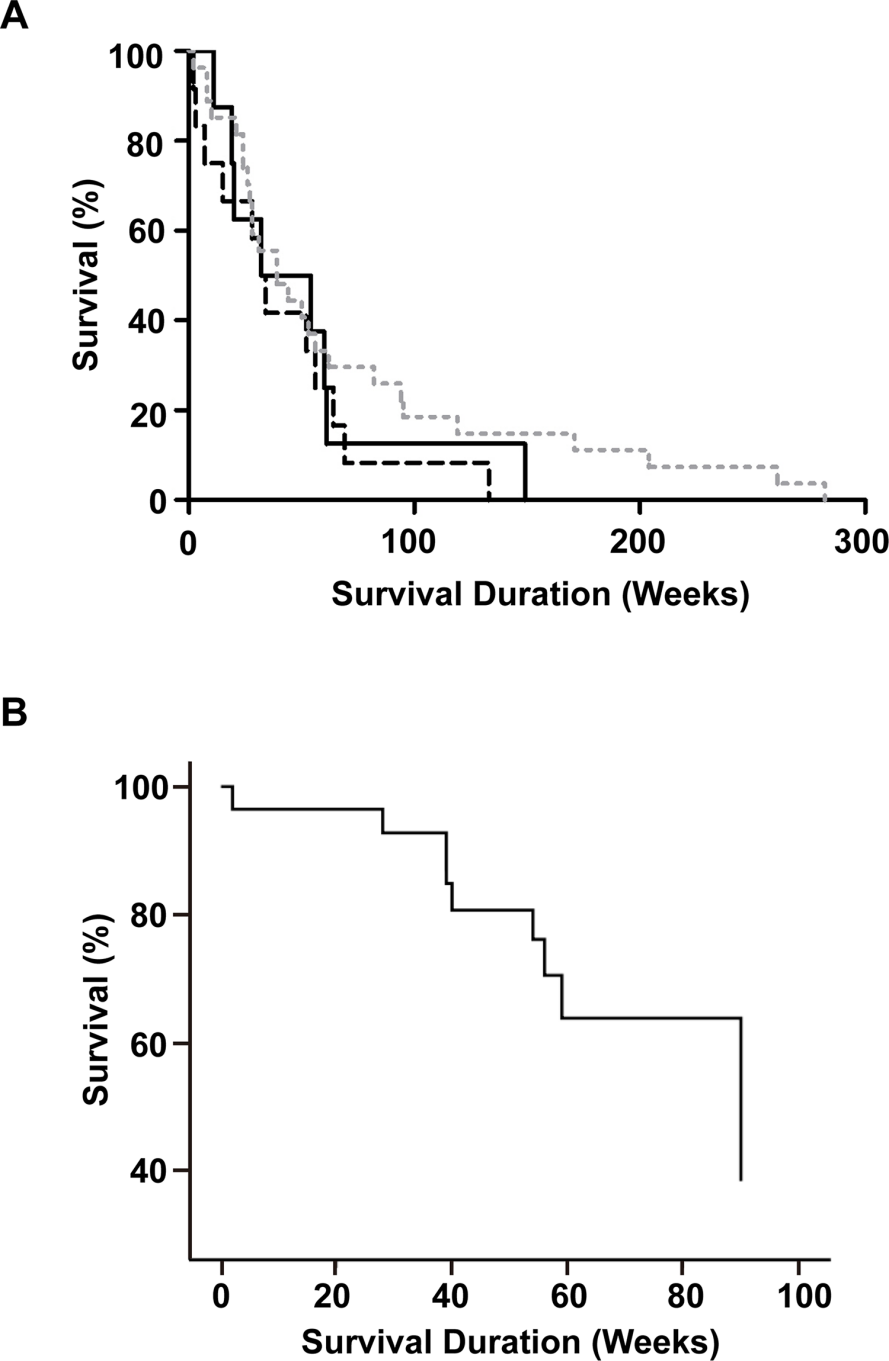
Correlation of *ZFPM2* genotype and expression with survival duration. (A) Survival duration profiles of different genotype groups for GBM. Black line, dashed line and grey dotted line represent homozygous insertion (II) genotype (n = 8, Male/Female = 1.66), heterozygous (ID) genotype (n = 12, Male/Female = 0.71) and homozygous deletion (DD) genotype (n = 27, Male/Female = 3.50) of rs71350512, respectively. The median survival time of the above three genotypes was 43 (range 11–149) weeks, 33 (range 2–133) weeks, and 39 (range 2–282) weeks, respectively. The *P* value based on the log rank test of Kaplan-Meier analysis was 0.339. (B) Correlation of *ZFPM2* expression with survival duration. The Cox regression plot for 15 glioma patients with *ZFPM2* expression level as covariate (exp(B) = 0.949; *P* = 0.077).

Upon follow up of 62 patients comprising 54 astrocytomas (including 50 GBMs), and 8 oligodendroglial tumors, 11 survived for a median of 91 (range 65–338) weeks, and 51 were deceased with a median survival of 40 (range 2–282) weeks. Based on the log rank test of Kaplan-Meier, there was no significant association between the genotypes and survival time of the 47 deceased GBM patients (*P* = 0.339) (Fig 5A).

The median survival time was 59 (range 2–98) weeks up to the study end point for 15 patients with expression data (median expression was 15.34; range 0.63–161.51). Cox regression analysis using expression level as covariate indicated that expression exerted a moderate ‘protective’ effect on survival (exp(B) = 0.949; *P* = 0.077) (Fig 5B).

### Natural selection acting on *ZFPM2*

**Fig 6:**
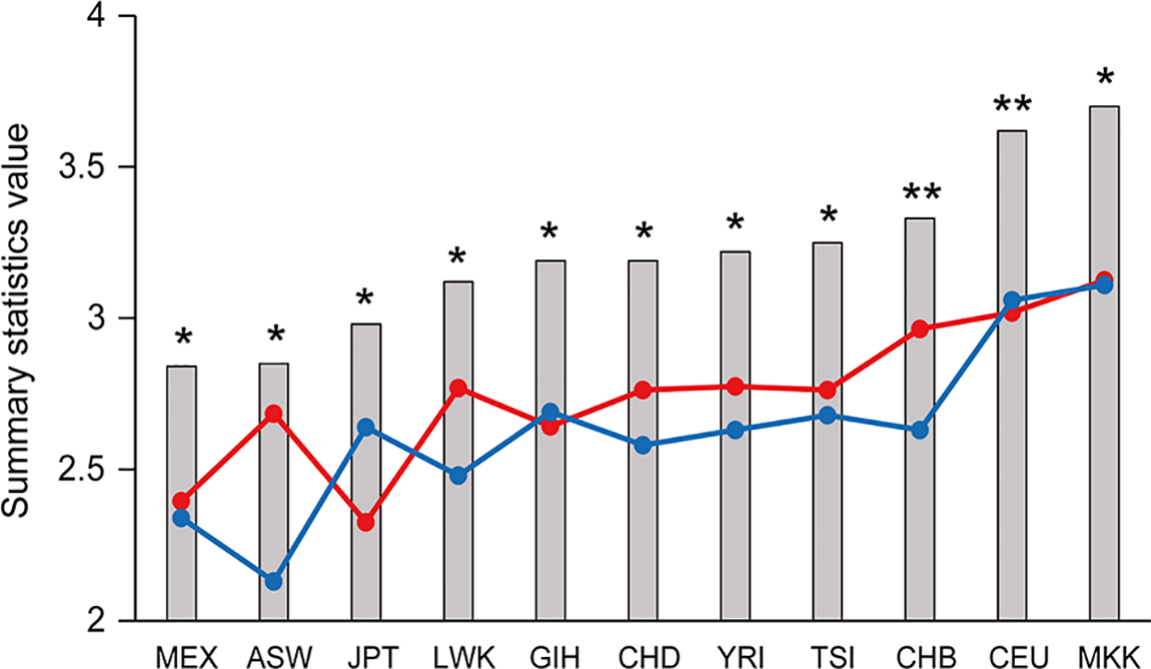
Summary statistics for *ZFPM2* are plotted for the 11 HapMap populations. The Tajima’s D (red circles), Fu and Li’s D* (blue circles) and Fu and Li’s F* (grey columns) values were obtained using HapMap Phase III data. The populations are ordered according to increasing Fu and Li’s F* values. All values significantly deviated from neutrality (*P* ≤ 0.01); * and ** depicts Tajima’s D values for which coalescent simulation gave *P* ≤ 0.01 and *P* < 0.001, respectively. The HapMap population descriptors are as follows: ASW: African ancestry in Southwest USA, CEU: Utah residents with Northern and Western European ancestry from the CEPH collection, CHB: Han Chinese in Beijing, China, CHD: Chinese in Metropolitan Denver, Colorado, GIH: Gujarati Indians in Houston, Texas, JPT: Japanese in Tokyo, Japan, LWK: Luhya in Webuye, Kenya, MEX: Mexican ancestry in Los Angeles, California, MKK: Maasai in Kinyawa, Kenya, TSI: Tuscan in Italy, YRI: Yoruban in Ibadan, Nigeria.

To investigate whether *ZFPM2* is under evolutionary pressure, data for all single nucleotide polymorphisms (SNPs) within the gene were retrieved from the HapMap Project database and used for summary statistics tests, including Tajima’s D, Fu and Li’s D* and Fu and Li’s F*, and these statistics were analyzed to determine whether they were significantly different from expected values under a standard neutral model by performing coalescent simulation assuming no recombination. The 11 populations sampled in Phase III of the HapMap Project all gave significantly positive values for the three summary statistics tested (Fig 6; S2 Table). Notably, populations of African, European and Asian ancestry were randomly ordered relative to the values of the summary statistics, suggesting there was no apparent correlation between the values and the evolutionary age of the populations.

## Discussion

### Genotyping marker selection

Previous studies have shown that Alu elements in the human genome can be used for mapping complicated disease alleles [37,38]. Thus the selection of rs71305152, close to both AluSp and AluSx within *ZFPM2*, as a potential glioma susceptibility marker in the present study is consistent with the frequent location of potential regions with disease-associated alleles in the neighborhood of Alu elements. In addition, its location within a large, highly-linked haplotype (Fig 1) makes it a suitable tagging marker representative of that region. Furthermore, the genotypes of the insertion-deletion polymorphism rs71305152 were found to be the same between the paired glioma-blood samples for thirty patients (data not shown), which indicated that rs71305152 is a potentially stable polymorphism locus without any loss of heterozygosity or somatic mutation. Although the two platforms of direct DNA sequencing and gel electrophoresis were both employed for genotyping in the present study to ensure the accuracy of the determined genotypes, notably the three *ZFPM2* genotypes were readily differentiated by simple gel electrophoresis alone. These properties render this potential glioma susceptibility marker suitable for clinical diagnostic purposes.

### Association between rs71305152 and gliomas

Association analysis showed that rs71305152 was strongly associated with gliomas (*P* = 0.016). This association was not previously identified in GWAS studies, possibly because the effective sample size of the polymorphism is too small for detection in such genome-wide approaches, and/or the six-nucleotide deletion itself was not included in the probe set in the array platforms employed in the GWAS. Nevertheless, the *P* value of Hardy-Weinberg equilibrium test, which has been validated as furnishing evidence for association [39], was dramatically less than 0.05 in the glioma cohort (*P* = 0.0015), thereby supporting *ZFPM2* as a glioma susceptibility gene. Compared to the control cohort, both the homozygous insertion genotype (*P* = 0.021) and the homozygous deletion genotype (*P* = 0.015) showed a moderate increase in gliomas (Table 3). Such frequency alterations of the two homozygous genotypes of a glioma-associated gene were observed for the first time in the present study, although similar changes in homozygous genotypes also have been encountered in other non-cancer disorders [40,41]. Moreover, the decrease in heterozygous genotype frequency in gliomas compared to either controls (*P* = 0.005), or non-glioma cancers (*P* = 0.020), suggests a protective role being played by the heterozygous genotype in gliomas and this is a characteristic of loci that are under the influence of balancing selection [27].

Interestingly, in Fig 3B the association between the heterozygous rs71305152 genotype and glioma was found to be significant only in male patients (*P* = 0.020), but not in female patients (*P* = 0.148). Epidemiological studies have shown a higher incidence of gliomas in males than females (male/female = 1.4–1.6) [42,43], and the possible involvement of an estrogen-protective effect has been suggested [44]. The present results suggest that the susceptibility gene *ZFPM2* could also be a contributing factor to the higher incidence of gliomas observed for males. Further studies with increased sample size would be most valuable in this regard.

As many susceptibility SNPs have been associated with more than one type of cancer [45], the present study also examined the relationships between rs71305152 and lung cancer, leukemia and lymphoma. Although *ZFPM2* plays an essential role in lung development [2], and the *ZFPM2*-related *FOG* gene is strongly expressed in immature hematopoietic cell lineages [46], Table 2 shows that no association was found between rs71305152 and lung cancer (*P* = 0.629), leukemia (*P* = 0.528) or lymphoma (*P* = 0.228). However, the explicit disassociation between rs71305152 and these cancer types need to be further tested with large sample sizes in the future. In addition, the possible association between this marker and more types of cancer need to be examined, especially for ovary tumor and neuroblastoma, where abnormal *ZFPM2* expression has been reported, in order to determine the extent of specificity of the rs71305152-glioma association.

### *ZFPM2* expression as a marker for severity and prognosis

Based on ANOVA analysis, the expression levels of *ZFPM2* were significantly different between the glioma grades (*P* = 0.012, Fig 4), and the astrocytoma subtype grades (*P* = 0.006, Table 4), which included the most severe grade IV GBM patients. These results suggest that disease severity could be prognostically correlated with reduced levels of *ZFPM2*. The finding of higher *ZFPM2* expression marginally increasing patient survival (exp(B) = 0.949; *P* = 0.077, Fig 5B) lends support to such a prognostic correlation. A comparable correlation between higher *ZFPM2* expression and spontaneous regression also has been reported for neuroblastoma [6].

Significant association was observed between patients’ genotypes and *ZFPM2* expression in astrocytomas (*P* = 0.028) as indicated in Fig 4C. However, there was no significant association between genotypes and grades in astrocytomas (data not shown) or survival time of the GBM cases (*P* = 0.339, Fig 5A), which indicated that the genotypes themselves are not useful prognostic markers. Therefore, the genotypes appear to exert their impact on the incidence of gliomas, possibly involving *ZFPM2* expression, but not the progression of glioma to severity. Nevertheless, all expression data was obtained from only a small sample set, and further studies with increased sample size are warranted.

### Balancing selection

Most human genetic mutations are neutral or deleterious, but some are advantageous and persist in the population either to fixation or maintained at high frequencies. The latter variations contribute to advantageous population diversity and are evolutionarily maintained by balancing selection [27]. A classic example is the sickle cell hemoglobin polymorphism where the homozygote individuals are either susceptible to malaria or sickle cell anemia while the heterozygotes are protected [47,48]. This heterozygote advantage is one of the underlying factors for balancing selection, and as such, balancing selection can be identified through the presence of a significant excess of heterozygotes or of higher fitness in heterozygotes. In the present study, the heterozygous genotype frequency for the indel rs71305152 in *ZFPM2* was revealed in Fig 3 to be significantly decreased in glioma patients compared to controls (*P* = 0.005), suggesting a potentially protective role played by the heterozygous genotype in gliomas. This is corroborated by the fact that the heterozygous genotype was the major genotype in the control cohort (46.65%), in contrast to its being a minor genotype in the glioma cohort (36.86%) as shown in Table 1, in accordance with the balancing selection theory where a heterozygote advantage can be observed in the population. The action of balancing selection on the gene was further examined with summary statistics tests, and the observation of highly positive values for *ZFPM2* for the three summary statistics Tajima’s D, Fu and Li’s D* and Fu and Li’s F* indeed indicated an excess of heterozygotes. Although this excess of intermediate-frequency alleles in a population can result from either balancing selection or population bottlenecks, given that these statistics were significantly positive across all the tested populations and not only to specific populations, it is unlikely for demographic effects to be a major factor. Both lines of evidence therefore provide strong evidence for balancing selection acting on the *ZFPM2* gene. Balancing selection is an important evolutionary force and its targets include genes in key functional systems such as the immune system and the reproductive system [28,49,50]. The current finding of balancing selection acting on *ZFPM2* may likewise be associated with its important roles in multiple organ development, including the reproductive organs.

## Conclusion

In summary, the present study represents the first instance where effects of *ZFPM2* on gliomas, the most prevalent brain tumor, and balancing selection in the gene have been examined. The genetic association results clearly indicate that rs71305152 is a susceptibility marker for gliomas in the *Han* Chinese population, especially for males. That the genotypes of *ZFPM2* could be a contributing factor to the greater prevalence of gliomas in males highlights the importance of the polymorphism. In addition, the relative stability of the polymorphism against somatic mutation and ease of its genotyping using gel electrophoresis underlies the potential of rs71305152 as a clinically useful marker. Moreover, *ZFPM2* expression levels were negatively correlated with glioma grades, suggesting that *ZFPM2* expression can be a potentially useful indicator of disease severity for gliomas, as well as a significant factor in glioma pathogenesis, in which case *ZFPM2* could provide a valuable target for glioma therapeutics. Finally, that balancing selection was found to act on *ZFPM2* highlights its evolutionary importance and further investigation is merited to understand how its fundamental functions contribute to its myriad involvement in organ development and disease phenotypes.

## Supporting Information

S1 Fig Genotype frequency distributions in different glioma subtypes (*P* = 0.973). A II and A III—IV represent low grade (II), and high grades (III—IV) astrocytomas. O + OA II represents grade II oligodendroglial tumors (oligodendrogliomas and anaplastic oligoastrocytomas). AO + AOA III represents grade III oligodendroglial tumors (anaplastic oligodendrogliomas and anaplastic oligoastrocytomas). Dark grey bars, light grey bars and white bars represent frequency of homozygous insertion (II), heterozygous (ID) and homozygous deletion (DD) genotypes, respectively. The sample size of each genotype is shown on each bar.
(PDF)

S1 Table Demographic characterization of the enrolled cohorts. (PDF)

S2 Table Summary statistics values for *ZFPM2* in the 11 HapMap populations. The values for the summary statistics Tajima’s D, Fu and Li’s D* and Fu and LI’s F* are given for the 11 HapMap populations. Nominal *P* values determined from 10^4^ coalescent simulations with no recombination are shown in the column next to each statistic.
(PDF)
