# Supplementary figures and images for: Glioma Association and Balancing Selection of ZFPM2
Source: PLoS One. 2015 Jul 24;10(7):e0133003. doi: 10.1371/journal.pone.0133003 (PMC4514883; doi:10.1371/journal.pone.0133003)

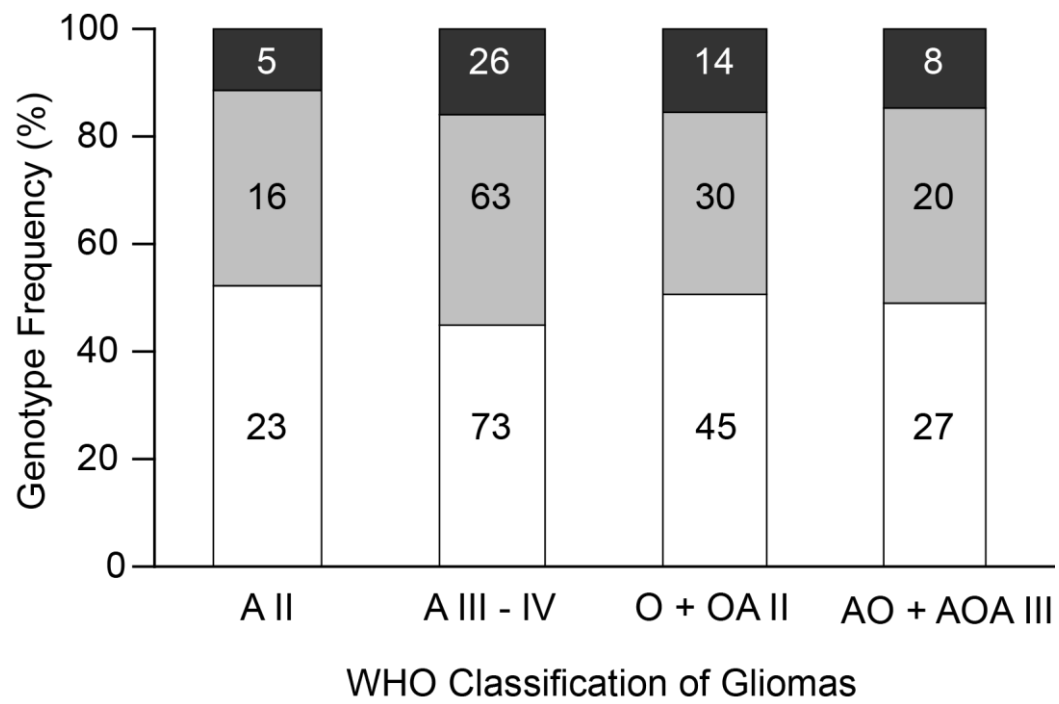

**S1 Fig. Genotype frequency distributions in different glioma subtypes ( $P = 0.973$ ).**

Supplement: S1 Fig — A II and A III—IV represent low grade (II), and high grades (III—IV) astrocytomas. O + OA II represents grade II oligodendroglial tumors (oligodendrogliomas and anaplastic oligoastrocytomas). AO + AOA III represents grade III oligodendroglial tumors (anaplastic oligodendrogliomas and anaplastic oligoastrocytomas). Dark grey bars, light grey bars and white bars represent frequency of homozygous insertion (II), heterozygous (ID) and homozygous deletion (DD) genotypes, respectively. The sample size of each genotype is shown on each bar. (PDF) [file pone.0133003.s001.pdf]
