# Supplementary material for: Glioma Association and Balancing Selection of ZFPM2
Source: PLoS One. 2015 Jul 24;10(7):e0133003. doi: 10.1371/journal.pone.0133003 (PMC4514883; doi:10.1371/journal.pone.0133003)
Supplement: S1 Table — (PDF) [file pone.0133003.s002.pdf]

S1 Table. Demographic characterization of the enrolled cohorts.

Cohort	Source	n	Male (%)	Female (%)	Age (yr) Mean ± SD
Glioma	Hong Kong	131	66.41	33.59	48.48 ±18.39
	Beijing	219	57.08	42.92	40.12 ±14.59
Lung Cancer	Guangzhou	109	67.89	32.11	54.42 ± 11.22
Lymphoma	Shanghai	129	56.59	43.41	51.49 ± 15.96
	Guangzhou	20	70.00	30.00	43.45 ± 17.19
Leukemia	Shanghai	97	53.61	46.39	38.96 ± 14.65
Control	Hong Kong	281	49.82	50.18	34.46 ± 10.18
	Beijing	186	51.61	48.39	N/A

N/A: not available
