# Supplementary material for: Glioma Association and Balancing Selection of ZFPM2
Source: PLoS One. 2015 Jul 24;10(7):e0133003. doi: 10.1371/journal.pone.0133003 (PMC4514883; doi:10.1371/journal.pone.0133003)
Supplement: S2 Table — The values for the summary statistics Tajima’s D, Fu and Li’s D* and Fu and LI’s F* are given for the 11 HapMap populations. Nominal P values determined from 104 coalescent simulations with no recombination are shown in the column next to each statistic. (PDF) [file pone.0133003.s003.pdf]

S2 Table. Summary statistics values for *ZFPM2* in the 11 HapMap populations.

Population	Tajima's D	P_D	Fu and Li's D*	P_{D*}	Fu and Li's F*	P_{F*}
ASW	2.68	0.002	2.13	<0.001	2.85	<0.001
CEU	3.02	<0.001	3.06	<0.001	3.62	<0.001
CHB	2.96	<0.001	2.63	<0.001	3.33	<0.001
CHD	2.76	0.004	2.58	<0.001	3.19	<0.001
GIH	2.64	0.004	2.69	<0.001	3.19	<0.001
JPT	2.32	0.010	2.64	<0.001	2.98	<0.001
LWK	2.77	0.003	2.48	<0.001	3.12	<0.001
MEX	2.40	0.005	2.34	<0.001	2.84	<0.001
MKK	3.12	0.002	3.11	<0.001	3.70	<0.001
TSI	2.76	0.003	2.68	<0.001	3.25	<0.001
YRI	2.78	0.003	2.63	<0.001	3.22	<0.001
